# Bis[4-(chloro­acet­yl)phenyl] ether

**DOI:** 10.1107/S1600536808021168

**Published:** 2008-07-16

**Authors:** Fan-Lei Meng, Liang-Zhong Xu

**Affiliations:** a College of Chemistry and Molecular Engineering, Qingdao University of Science and Technology, Qingdao 266042, People’s Republic of China

## Abstract

The title compound, C_16_H_12_Cl_2_O_3_, crystallizes with two independent mol­ecules in the asymmetric unit. The dihedral angles between the planes of the benzene rings in the two independent mol­ecules are 68.65 (2) and 68.47 (3)°. The short distance of 3.899 (5) Å between the centroids of the benzene rings of neighbouring mol­ecules indicate π–π inter­actions. The crystal structure is stabilized by a network of intermolecular C—H⋯O hydrogen bonds.

## Related literature

For biological activity, see: Fujimoto & Quinn (1988 ▶). For similar structures, see: Grossert *et al.* (1984 ▶). For the preparation, see: Edward & Sibelle (1963 ▶).
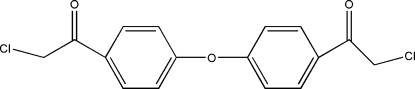


## Experimental

### 

#### Crystal data

C_16_H_12_Cl_2_O_3_
*M* *_r_* = 323.16
Monoclinic, 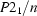

*a* = 12.597 (3) Å
*b* = 9.2042 (18) Å
*c* = 25.320 (5) Å
β = 104.18 (3)°
*V* = 2846.3 (10) Å^3^
*Z* = 8
Mo *K*α radiation
μ = 0.46 mm^−1^
*T* = 113 (2) K
0.24 × 0.18 × 0.04 mm

#### Data collection

Rigaku Saturn diffractometer
Absorption correction: multi-scan (*CrystalClear*; Rigaku, 2005 ▶) *T*_min_ = 0.897, *T*_max_ = 0.982
20200 measured reflections
6778 independent reflections
5751 reflections with *I* > 2σ(*I*)
*R*_int_ = 0.039

#### Refinement

*R*[*F* ^2^ > 2σ(*F* ^2^)] = 0.040
*wR*(*F* ^2^) = 0.109
*S* = 1.09
6778 reflections
379 parameters
H-atom parameters constrained
Δρ_max_ = 0.64 e Å^−3^
Δρ_min_ = −0.71 e Å^−3^

### 

Data collection: *CrystalClear* (Rigaku, 2005 ▶); cell refinement: *CrystalClear*; data reduction: *CrystalClear*; program(s) used to solve structure: *SHELXTL* (Sheldrick, 2008 ▶); program(s) used to refine structure: *SHELXTL*; molecular graphics: *SHELXTL*; software used to prepare material for publication: *SHELXTL*.

## Supplementary Material

Crystal structure: contains datablocks I, global. DOI: 10.1107/S1600536808021168/hg2419sup1.cif

Structure factors: contains datablocks I. DOI: 10.1107/S1600536808021168/hg2419Isup2.hkl

Additional supplementary materials:  crystallographic information; 3D view; checkCIF report

## Figures and Tables

**Table 1:** Hydrogen-bond geometry (Å, °)

*D*—H⋯*A*	*D*—H	H⋯*A*	*D*⋯*A*	*D*—H⋯*A*
C1—H1*B*⋯O3^ii^	0.97	2.24	3.103 (2)	147
C10—H10⋯O4^iii^	0.93	2.42	3.286 (2)	155
C14—H14⋯O6^iv^	0.93	2.38	3.242 (2)	154
C17—H17*A*⋯O4^v^	0.97	2.29	3.255 (2)	173
C21—H21⋯O1^vi^	0.93	2.54	3.469 (2)	176

Symmetry codes: (ii) 
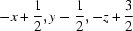
; (iii) 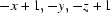; (iv) 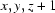; (v) 
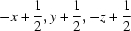
; (vi) 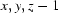.

**Fig. 1.**
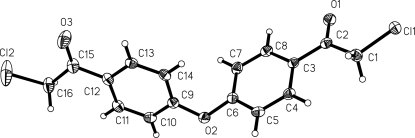
View of the first molecule of the title compound (I), with displacement ellipsoids drawn at the 50% probability level.

**Fig. 2.**
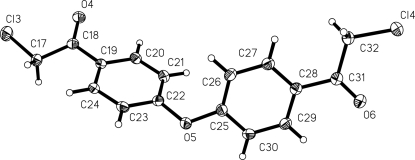
View of the second molecule of the title compound (I), with displacement ellipsoids drawn at the 50% probability level.

**Fig. 3.**
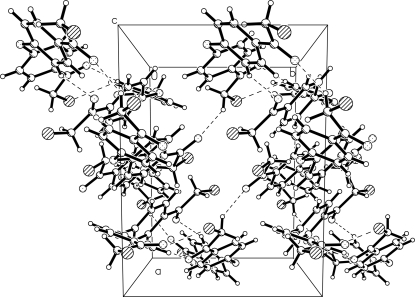
A packing diagram of the molecule of the title compound, viewed down c axis. Hydrogen bonds are shown as dashed lines.

## supplementary crystallographic information

### Comment

The title compound, was obtained unintentionally  as an intermediate for the synthesis of Triazole compounds. Triazole compounds had been found to show wide spread biological activities. Many of them had been developed and used as fungicides, plant growth regulators and medicine. (Fujimoto & Quinn, 1988) we report here the crystal structure of (I).

The title compound, crystallizes in space group with two independent molecules in the asymmetric unit (Figs. 1,2). All bond lengths and angles are normal and in a good agreement with those reported previously (Grossert *et al.*, 1984). The angles of C6—O2—C9 and C22—O15—C25 in the two independent molecules are 119.06 (2) and 120.07 (3)°.  However, the two benzene rings are not coplanar planar. The dihedral angles between the planes of benzene rings in the two independent molecules are 68.65 (2) and 68.47 (3)°. π-π stacking interactions are present in the structure. The crystal structure is stabilized by a network of hydrogen bonds and van der Waals interations.

### Experimental

A mixture of 1-phenoxybenzene(5.0 mmol) and anhydrous aluminium chloride were added to a solution of 50 mL of dry dichloromethane in a flask equipped with stirrer and reflux condenser. Chloroacetyl chloride (10.0 mmol) was slowly added from a dropping-funnel to the boiling mixture during 30 minutes After this addition, the reaction mixture was heated with strring for two hours at boiling. The mixture was poured into ice-water and extracted with dichloromethane. The extract was dried over anhydrous magnesium sulfate, and dichloromethane was distilled off. The residue was purified by a column chromatography to obtain the title compound (10.1 g, yield 62%).(Edward & Sibelle, 1963). Single crystals suitable for X-ray measurement were obtained by recrystallization from petroleum ether at room temperature. mp.383-384 K.

### Refinement

All H atoms were found on difference maps, H atoms were placed in calculated positions, with C—H = 0.93 or 0.97Å, and included in the final cycles of refinement using a riding model, with *U*_iso_(H) = 1.2 times *U*_eq_(C).

### Crystal data

**Table:**

C_16_H_12_Cl_2_O_3_	*F*_000_ = 1328
*M**_r_* = 323.16	*D*_x_ = 1.508 Mg m^−^^3^
Monoclinic, *P*2_1_/*n*	Mo *K*α radiation λ = 0.71073 Å
Hall symbol: -P 2yn	Cell parameters from 7912 reflections
*a* = 12.597 (3) Å	θ = 1.7–27.9º
*b* = 9.2042 (18) Å	µ = 0.46 mm^−^^1^
*c* = 25.320 (5) Å	*T* = 113 (2) K
β = 104.18 (3)º	Platelet, colorless
*V* = 2846.3 (10) Å^3^	0.24 × 0.18 × 0.04 mm
*Z* = 8	

### Data collection

**Table:**

Rigaku Saturn diffractometer	6778 independent reflections
Radiation source: Rotating Anode	5751 reflections with *I* > 2σ(*I*)
Monochromator: confocal	*R*_int_ = 0.039
*T* = 113(2) K	θ_max_ = 27.9º
ω scans	θ_min_ = 1.7º
Absorption correction: multi-scan(CrystalClear; Rigaku, 2005)	*h* = −16→16
*T*_min_ = 0.897, *T*_max_ = 0.982	*k* = −12→12
20200 measured reflections	*l* = −19→33

### Refinement

**Table:**

Refinement on *F*^2^	Secondary atom site location: difference Fourier map
Least-squares matrix: full	Hydrogen site location: inferred from neighbouring sites
*R*[*F*^2^ > 2σ(*F*^2^)] = 0.040	H-atom parameters constrained
*wR*(*F*^2^) = 0.109	' *w* = 1/[σ^2^(*F*_o_^2^) + (0.0573*P*)^2^ + 0.5125*P*] where *P* = (*F*_o_^2^ + 2*F*_c_^2^)/3
*S* = 1.09	(Δ/σ)_max_ = 0.002
6778 reflections	Δρ_max_ = 0.64 e Å^−^^3^
379 parameters	Δρ_min_ = −0.71 e Å^−^^3^
Primary atom site location: structure-invariant direct methods	Extinction correction: none

### Special details

**Table:**

Geometry. All e.s.d.'s (except the e.s.d. in the dihedral angle between two l.s. planes) are estimated using the full covariance matrix. The cell e.s.d.'s are taken into account individually in the estimation of e.s.d.'s in distances, angles and torsion angles; correlations between e.s.d.'s in cell parameters are only used when they are defined by crystal symmetry. An approximate (isotropic) treatment of cell e.s.d.'s is used for estimating e.s.d.'s involving l.s. planes.
Refinement. Refinement of *F*^2^ against ALL reflections. The weighted *R*-factor *wR* and goodness of fit *S* are based on *F*^2^, conventional *R*-factors *R* are based on *F*, with *F* set to zero for negative *F*^2^. The threshold expression of *F*^2^ > σ(*F*^2^) is used only for calculating *R*-factors(gt) *etc*. and is not relevant to the choice of reflections for refinement. *R*-factors based on *F*^2^ are statistically about twice as large as those based on *F*, and *R*- factors based on ALL data will be even larger.

### Fractional atomic coordinates and isotropic or equivalent isotropic displacement parameters (Å^2^)

**Table:**

	*x*	*y*	*z*	*U*_iso_*/*U*_eq_	
Cl1	0.28427 (4)	0.08039 (4)	1.058487 (17)	0.02603 (11)	
Cl2	0.47207 (5)	0.29171 (9)	0.48766 (2)	0.0642 (2)	
Cl3	0.35388 (5)	0.50683 (6)	0.343823 (18)	0.04085 (14)	
Cl4	0.12503 (4)	0.05428 (4)	−0.234906 (17)	0.02692 (11)	
O1	0.43216 (10)	0.20625 (13)	0.99868 (5)	0.0249 (3)	
O2	0.48320 (10)	−0.08423 (12)	0.77943 (5)	0.0242 (3)	
O3	0.38823 (12)	0.35813 (17)	0.58187 (6)	0.0420 (4)	
O4	0.33203 (11)	0.32837 (12)	0.24803 (5)	0.0265 (3)	
O5	0.31588 (11)	0.62621 (12)	0.02015 (5)	0.0267 (3)	
O6	0.26773 (10)	0.30081 (13)	−0.20407 (5)	0.0262 (3)	
C1	0.30788 (15)	0.00873 (19)	0.99746 (7)	0.0245 (4)	
H1A	0.3371	−0.0889	1.0043	0.029*	
H1B	0.2387	0.0023	0.9703	0.029*	
C2	0.38694 (13)	0.10026 (17)	0.97496 (6)	0.0190 (3)	
C3	0.40671 (13)	0.05153 (16)	0.92204 (6)	0.0182 (3)	
C4	0.36589 (14)	−0.07866 (17)	0.89682 (7)	0.0217 (3)	
H4	0.3221	−0.1374	0.9126	0.026*	
C5	0.38986 (14)	−0.12143 (18)	0.84859 (7)	0.0225 (3)	
H5	0.3630	−0.2087	0.8321	0.027*	
C6	0.45426 (13)	−0.03237 (17)	0.82532 (7)	0.0199 (3)	
C7	0.49463 (14)	0.09832 (18)	0.84929 (7)	0.0235 (3)	
H7	0.5371	0.1577	0.8329	0.028*	
C8	0.47138 (13)	0.13953 (18)	0.89752 (7)	0.0219 (3)	
H8	0.4989	0.2266	0.9139	0.026*	
C9	0.47682 (13)	0.00766 (17)	0.73577 (7)	0.0203 (3)	
C10	0.54686 (13)	−0.02410 (18)	0.70265 (7)	0.0226 (3)	
H10	0.5982	−0.0983	0.7120	0.027*	
C11	0.53955 (13)	0.05576 (18)	0.65557 (7)	0.0225 (3)	
H11	0.5858	0.0345	0.6331	0.027*	
C12	0.46343 (13)	0.16782 (18)	0.64155 (7)	0.0209 (3)	
C13	0.39497 (13)	0.19865 (18)	0.67615 (7)	0.0210 (3)	
H13	0.3445	0.2740	0.6673	0.025*	
C14	0.40084 (13)	0.11975 (17)	0.72302 (7)	0.0209 (3)	
H14	0.3549	0.1410	0.7457	0.025*	
C15	0.44999 (14)	0.2549 (2)	0.59125 (7)	0.0268 (4)	
C16	0.51660 (15)	0.2091 (2)	0.55157 (7)	0.0301 (4)	
H16A	0.5120	0.1045	0.5471	0.036*	
H16B	0.5928	0.2339	0.5668	0.036*	
C17	0.35304 (14)	0.57407 (18)	0.27839 (6)	0.0223 (3)	
H17A	0.2951	0.6455	0.2678	0.027*	
H17B	0.4221	0.6224	0.2798	0.027*	
C18	0.33593 (12)	0.45517 (17)	0.23596 (6)	0.0188 (3)	
C19	0.32804 (12)	0.50180 (16)	0.17890 (6)	0.0173 (3)	
C20	0.35688 (13)	0.40140 (17)	0.14305 (7)	0.0203 (3)	
H20	0.3796	0.3087	0.1554	0.024*	
C21	0.35203 (14)	0.43836 (17)	0.08969 (7)	0.0221 (3)	
H21	0.3728	0.3723	0.0663	0.027*	
C22	0.31543 (14)	0.57643 (17)	0.07159 (6)	0.0206 (3)	
C23	0.28435 (14)	0.67673 (17)	0.10592 (7)	0.0211 (3)	
H23	0.2584	0.7678	0.0929	0.025*	
C24	0.29241 (13)	0.63979 (17)	0.15968 (6)	0.0188 (3)	
H24	0.2739	0.7075	0.1832	0.023*	
C25	0.29220 (14)	0.53207 (17)	−0.02388 (6)	0.0211 (3)	
C26	0.20681 (14)	0.43327 (18)	−0.03160 (7)	0.0222 (3)	
H26	0.1665	0.4231	−0.0056	0.027*	
C27	0.18197 (13)	0.34955 (18)	−0.07870 (7)	0.0206 (3)	
H27	0.1252	0.2824	−0.0840	0.025*	
C28	0.24164 (13)	0.36558 (17)	−0.11809 (6)	0.0183 (3)	
C29	0.32770 (14)	0.46592 (17)	−0.10904 (7)	0.0214 (3)	
H29	0.3678	0.4774	−0.1351	0.026*	
C30	0.35398 (14)	0.54819 (17)	−0.06193 (7)	0.0221 (3)	
H30	0.4122	0.6133	−0.0559	0.027*	
C31	0.21875 (13)	0.27894 (17)	−0.16926 (6)	0.0189 (3)	
C32	0.13163 (14)	0.16104 (18)	−0.17589 (7)	0.0223 (3)	
H32A	0.0609	0.2059	−0.1783	0.027*	
H32B	0.1479	0.0987	−0.1440	0.027*	

### Atomic displacement parameters (Å^2^)

**Table:**

	*U*^11^	*U*^22^	*U*^33^	*U*^12^	*U*^13^	*U*^23^
Cl1	0.0326 (2)	0.0240 (2)	0.0234 (2)	−0.00095 (16)	0.01054 (17)	−0.00148 (16)
Cl2	0.0477 (3)	0.1203 (6)	0.0283 (3)	0.0339 (4)	0.0166 (2)	0.0261 (3)
Cl3	0.0682 (4)	0.0373 (3)	0.0204 (2)	−0.0108 (2)	0.0173 (2)	−0.00209 (18)
Cl4	0.0334 (2)	0.0237 (2)	0.0241 (2)	−0.00443 (17)	0.00773 (17)	−0.00560 (15)
O1	0.0278 (6)	0.0218 (6)	0.0239 (6)	−0.0053 (5)	0.0036 (5)	−0.0020 (5)
O2	0.0288 (6)	0.0204 (6)	0.0260 (6)	0.0025 (5)	0.0118 (5)	0.0015 (5)
O3	0.0343 (8)	0.0576 (9)	0.0377 (8)	0.0234 (7)	0.0157 (6)	0.0208 (7)
O4	0.0370 (7)	0.0181 (6)	0.0255 (6)	−0.0017 (5)	0.0100 (5)	0.0027 (5)
O5	0.0445 (8)	0.0182 (6)	0.0190 (6)	−0.0065 (5)	0.0109 (5)	−0.0015 (5)
O6	0.0318 (7)	0.0273 (6)	0.0222 (6)	−0.0031 (5)	0.0120 (5)	−0.0025 (5)
C1	0.0286 (9)	0.0235 (8)	0.0221 (8)	−0.0047 (7)	0.0077 (7)	−0.0036 (7)
C2	0.0172 (7)	0.0181 (7)	0.0190 (8)	0.0023 (6)	−0.0005 (6)	0.0026 (6)
C3	0.0175 (7)	0.0171 (7)	0.0184 (7)	0.0019 (6)	0.0016 (6)	0.0023 (6)
C4	0.0212 (8)	0.0189 (8)	0.0256 (8)	−0.0034 (6)	0.0071 (7)	0.0015 (6)
C5	0.0223 (8)	0.0166 (8)	0.0284 (9)	−0.0013 (6)	0.0061 (7)	−0.0017 (6)
C6	0.0182 (8)	0.0211 (8)	0.0205 (8)	0.0028 (6)	0.0050 (6)	0.0014 (6)
C7	0.0237 (8)	0.0237 (8)	0.0233 (8)	−0.0055 (7)	0.0058 (7)	0.0026 (7)
C8	0.0211 (8)	0.0193 (8)	0.0227 (8)	−0.0027 (6)	0.0005 (6)	0.0008 (6)
C9	0.0195 (8)	0.0204 (8)	0.0209 (8)	−0.0024 (6)	0.0049 (6)	−0.0016 (6)
C10	0.0178 (8)	0.0221 (8)	0.0284 (9)	0.0030 (6)	0.0064 (7)	−0.0012 (7)
C11	0.0169 (8)	0.0281 (9)	0.0242 (8)	0.0006 (6)	0.0081 (6)	−0.0033 (7)
C12	0.0166 (8)	0.0260 (8)	0.0195 (8)	−0.0009 (6)	0.0031 (6)	−0.0029 (6)
C13	0.0172 (8)	0.0206 (8)	0.0242 (8)	0.0022 (6)	0.0033 (6)	−0.0031 (6)
C14	0.0186 (8)	0.0229 (8)	0.0224 (8)	−0.0007 (6)	0.0072 (6)	−0.0041 (6)
C15	0.0177 (8)	0.0386 (10)	0.0235 (9)	0.0038 (7)	0.0040 (6)	0.0021 (7)
C16	0.0253 (9)	0.0436 (11)	0.0223 (9)	0.0040 (8)	0.0077 (7)	0.0036 (8)
C17	0.0280 (9)	0.0234 (8)	0.0157 (7)	−0.0015 (7)	0.0052 (6)	0.0001 (6)
C18	0.0149 (7)	0.0201 (8)	0.0211 (8)	−0.0007 (6)	0.0038 (6)	0.0005 (6)
C19	0.0169 (7)	0.0173 (7)	0.0179 (7)	−0.0035 (6)	0.0050 (6)	−0.0017 (6)
C20	0.0223 (8)	0.0160 (7)	0.0223 (8)	0.0005 (6)	0.0047 (6)	−0.0008 (6)
C21	0.0255 (8)	0.0194 (8)	0.0222 (8)	−0.0011 (6)	0.0076 (7)	−0.0040 (6)
C22	0.0236 (8)	0.0209 (8)	0.0175 (7)	−0.0063 (6)	0.0053 (6)	−0.0004 (6)
C23	0.0253 (8)	0.0155 (7)	0.0222 (8)	−0.0025 (6)	0.0056 (7)	0.0003 (6)
C24	0.0213 (8)	0.0155 (7)	0.0205 (8)	−0.0027 (6)	0.0072 (6)	−0.0022 (6)
C25	0.0292 (9)	0.0168 (7)	0.0166 (8)	0.0010 (6)	0.0043 (6)	0.0006 (6)
C26	0.0255 (9)	0.0235 (8)	0.0195 (8)	−0.0005 (7)	0.0093 (7)	0.0013 (6)
C27	0.0201 (8)	0.0210 (8)	0.0204 (8)	−0.0006 (6)	0.0046 (6)	0.0014 (6)
C28	0.0190 (8)	0.0175 (7)	0.0179 (8)	0.0030 (6)	0.0037 (6)	0.0026 (6)
C29	0.0259 (9)	0.0197 (8)	0.0202 (8)	0.0000 (6)	0.0089 (7)	0.0020 (6)
C30	0.0251 (9)	0.0186 (8)	0.0228 (8)	−0.0041 (6)	0.0062 (7)	0.0021 (6)
C31	0.0195 (8)	0.0182 (7)	0.0189 (8)	0.0045 (6)	0.0044 (6)	0.0020 (6)
C32	0.0245 (9)	0.0220 (8)	0.0213 (8)	−0.0001 (6)	0.0072 (7)	−0.0025 (6)

### Geometric parameters (Å, °)

**Table:**

Cl1—C1	1.7711 (17)	C13—C14	1.378 (2)
Cl2—C16	1.7511 (19)	C13—H13	0.9300
Cl3—C17	1.7661 (17)	C14—H14	0.9300
Cl4—C32	1.7732 (17)	C15—C16	1.518 (2)
O1—C2	1.212 (2)	C16—H16A	0.9700
O2—C9	1.379 (2)	C16—H16B	0.9700
O2—C6	1.385 (2)	C17—C18	1.512 (2)
O3—C15	1.214 (2)	C17—H17A	0.9700
O4—C18	1.2105 (19)	C17—H17B	0.9700
O5—C22	1.3821 (19)	C18—C19	1.487 (2)
O5—C25	1.3854 (19)	C19—C24	1.394 (2)
O6—C31	1.2104 (19)	C19—C20	1.404 (2)
C1—C2	1.518 (2)	C20—C21	1.380 (2)
C1—H1A	0.9700	C20—H20	0.9300
C1—H1B	0.9700	C21—C22	1.391 (2)
C2—C3	1.491 (2)	C21—H21	0.9300
C3—C4	1.395 (2)	C22—C23	1.389 (2)
C3—C8	1.397 (2)	C23—C24	1.382 (2)
C4—C5	1.385 (2)	C23—H23	0.9300
C4—H4	0.9300	C24—H24	0.9300
C5—C6	1.383 (2)	C25—C26	1.385 (2)
C5—H5	0.9300	C25—C30	1.387 (2)
C6—C7	1.387 (2)	C26—C27	1.390 (2)
C7—C8	1.377 (2)	C26—H26	0.9300
C7—H7	0.9300	C27—C28	1.396 (2)
C8—H8	0.9300	C27—H27	0.9300
C9—C10	1.389 (2)	C28—C29	1.400 (2)
C9—C14	1.390 (2)	C28—C31	1.488 (2)
C10—C11	1.384 (2)	C29—C30	1.383 (2)
C10—H10	0.9300	C29—H29	0.9300
C11—C12	1.394 (2)	C30—H30	0.9300
C11—H11	0.9300	C31—C32	1.523 (2)
C12—C13	1.401 (2)	C32—H32A	0.9700
C12—C15	1.479 (2)	C32—H32B	0.9700
			
Cg2···Cg4^i^	3.899 (5)		
			
C9—O2—C6	119.06 (12)	H16A—C16—H16B	107.8
C22—O5—C25	120.07 (12)	C18—C17—Cl3	112.34 (12)
C2—C1—Cl1	112.74 (12)	C18—C17—H17A	109.1
C2—C1—H1A	109.0	Cl3—C17—H17A	109.1
Cl1—C1—H1A	109.0	C18—C17—H17B	109.1
C2—C1—H1B	109.0	Cl3—C17—H17B	109.1
Cl1—C1—H1B	109.0	H17A—C17—H17B	107.9
H1A—C1—H1B	107.8	O4—C18—C19	121.77 (14)
O1—C2—C3	121.72 (15)	O4—C18—C17	121.73 (15)
O1—C2—C1	122.19 (15)	C19—C18—C17	116.46 (13)
C3—C2—C1	116.09 (14)	C24—C19—C20	119.07 (14)
C4—C3—C8	118.95 (15)	C24—C19—C18	122.77 (14)
C4—C3—C2	123.07 (14)	C20—C19—C18	118.15 (14)
C8—C3—C2	117.96 (14)	C21—C20—C19	120.95 (15)
C5—C4—C3	120.80 (15)	C21—C20—H20	119.5
C5—C4—H4	119.6	C19—C20—H20	119.5
C3—C4—H4	119.6	C20—C21—C22	118.65 (15)
C6—C5—C4	118.99 (15)	C20—C21—H21	120.7
C6—C5—H5	120.5	C22—C21—H21	120.7
C4—C5—H5	120.5	O5—C22—C23	115.89 (14)
C5—C6—O2	117.11 (14)	O5—C22—C21	122.41 (14)
C5—C6—C7	121.22 (15)	C23—C22—C21	121.51 (15)
O2—C6—C7	121.50 (14)	C24—C23—C22	119.28 (15)
C8—C7—C6	119.49 (15)	C24—C23—H23	120.4
C8—C7—H7	120.3	C22—C23—H23	120.4
C6—C7—H7	120.3	C23—C24—C19	120.50 (14)
C7—C8—C3	120.54 (16)	C23—C24—H24	119.8
C7—C8—H8	119.7	C19—C24—H24	119.8
C3—C8—H8	119.7	O5—C25—C26	122.06 (15)
O2—C9—C10	115.73 (14)	O5—C25—C30	116.43 (15)
O2—C9—C14	122.89 (14)	C26—C25—C30	121.37 (15)
C10—C9—C14	121.25 (15)	C25—C26—C27	119.27 (15)
C11—C10—C9	119.34 (15)	C25—C26—H26	120.4
C11—C10—H10	120.3	C27—C26—H26	120.4
C9—C10—H10	120.3	C26—C27—C28	120.47 (15)
C10—C11—C12	120.59 (15)	C26—C27—H27	119.8
C10—C11—H11	119.7	C28—C27—H27	119.8
C12—C11—H11	119.7	C27—C28—C29	118.93 (15)
C11—C12—C13	118.76 (15)	C27—C28—C31	122.82 (15)
C11—C12—C15	123.15 (15)	C29—C28—C31	118.24 (14)
C13—C12—C15	118.08 (15)	C30—C29—C28	120.97 (15)
C14—C13—C12	121.37 (15)	C30—C29—H29	119.5
C14—C13—H13	119.3	C28—C29—H29	119.5
C12—C13—H13	119.3	C29—C30—C25	118.96 (15)
C13—C14—C9	118.67 (15)	C29—C30—H30	120.5
C13—C14—H14	120.7	C25—C30—H30	120.5
C9—C14—H14	120.7	O6—C31—C28	121.36 (15)
O3—C15—C12	121.69 (15)	O6—C31—C32	121.35 (14)
O3—C15—C16	121.29 (16)	C28—C31—C32	117.29 (13)
C12—C15—C16	117.02 (15)	C31—C32—Cl4	111.81 (11)
C15—C16—Cl2	112.78 (13)	C31—C32—H32A	109.3
C15—C16—H16A	109.0	Cl4—C32—H32A	109.3
Cl2—C16—H16A	109.0	C31—C32—H32B	109.3
C15—C16—H16B	109.0	Cl4—C32—H32B	109.3
Cl2—C16—H16B	109.0	H32A—C32—H32B	107.9
			
Cl1—C1—C2—O1	3.6 (2)	Cl3—C17—C18—O4	−5.2 (2)
Cl1—C1—C2—C3	−176.92 (11)	Cl3—C17—C18—C19	176.87 (11)
O1—C2—C3—C4	172.50 (15)	O4—C18—C19—C24	155.76 (16)
C1—C2—C3—C4	−7.0 (2)	C17—C18—C19—C24	−26.3 (2)
O1—C2—C3—C8	−6.0 (2)	O4—C18—C19—C20	−23.6 (2)
C1—C2—C3—C8	174.55 (15)	C17—C18—C19—C20	154.32 (15)
C8—C3—C4—C5	0.8 (2)	C24—C19—C20—C21	1.0 (2)
C2—C3—C4—C5	−177.69 (15)	C18—C19—C20—C21	−179.55 (15)
C3—C4—C5—C6	−0.6 (2)	C19—C20—C21—C22	−1.5 (2)
C4—C5—C6—O2	175.19 (14)	C25—O5—C22—C23	−148.34 (16)
C4—C5—C6—C7	−0.2 (3)	C25—O5—C22—C21	36.6 (2)
C9—O2—C6—C5	135.95 (15)	C20—C21—C22—O5	174.95 (15)
C9—O2—C6—C7	−48.7 (2)	C20—C21—C22—C23	0.2 (2)
C5—C6—C7—C8	0.8 (3)	O5—C22—C23—C24	−173.52 (14)
O2—C6—C7—C8	−174.35 (15)	C21—C22—C23—C24	1.6 (2)
C6—C7—C8—C3	−0.7 (3)	C22—C23—C24—C19	−2.0 (2)
C4—C3—C8—C7	−0.1 (2)	C20—C19—C24—C23	0.7 (2)
C2—C3—C8—C7	178.42 (15)	C18—C19—C24—C23	−178.62 (14)
C6—O2—C9—C10	153.50 (15)	C22—O5—C25—C26	43.4 (2)
C6—O2—C9—C14	−30.5 (2)	C22—O5—C25—C30	−140.70 (16)
O2—C9—C10—C11	174.92 (14)	O5—C25—C26—C27	175.11 (15)
C14—C9—C10—C11	−1.1 (2)	C30—C25—C26—C27	−0.6 (3)
C9—C10—C11—C12	0.5 (3)	C25—C26—C27—C28	−0.6 (2)
C10—C11—C12—C13	0.4 (2)	C26—C27—C28—C29	0.8 (2)
C10—C11—C12—C15	−178.62 (16)	C26—C27—C28—C31	179.98 (15)
C11—C12—C13—C14	−0.7 (2)	C27—C28—C29—C30	0.1 (2)
C15—C12—C13—C14	178.35 (15)	C31—C28—C29—C30	−179.09 (15)
C12—C13—C14—C9	0.1 (2)	C28—C29—C30—C25	−1.3 (2)
O2—C9—C14—C13	−174.94 (14)	O5—C25—C30—C29	−174.43 (14)
C10—C9—C14—C13	0.8 (2)	C26—C25—C30—C29	1.5 (3)
C11—C12—C15—O3	−174.96 (18)	C27—C28—C31—O6	175.80 (15)
C13—C12—C15—O3	6.0 (3)	C29—C28—C31—O6	−5.0 (2)
C11—C12—C15—C16	5.3 (3)	C27—C28—C31—C32	−4.5 (2)
C13—C12—C15—C16	−173.72 (16)	C29—C28—C31—C32	174.64 (14)
O3—C15—C16—Cl2	−13.4 (3)	O6—C31—C32—Cl4	6.4 (2)
C12—C15—C16—Cl2	166.39 (13)	C28—C31—C32—Cl4	−173.22 (11)

Symmetry codes: (i) *x*−1/2, −*y*+1/2, *z*−1/2.

### Hydrogen-bond geometry (Å, °)

**Table:**

*D*—H···*A*	*D*—H	H···*A*	*D*···*A*	*D*—H···*A*
C1—H1B···O3^ii^	0.97	2.24	3.103 (2)	147
C10—H10···O4^iii^	0.93	2.42	3.286 (2)	155
C14—H14···O6^iv^	0.93	2.38	3.242 (2)	154
C17—H17A···O4^v^	0.97	2.29	3.255 (2)	173
C21—H21···O1^vi^	0.93	2.54	3.469 (2)	176

Symmetry codes: (ii) −*x*+1/2, *y*−1/2, −*z*+3/2; (iii) −*x*+1, −*y*, −*z*+1; (iv) *x*, *y*, *z*+1; (v) −*x*+1/2, *y*+1/2, −*z*+1/2; (vi) *x*, *y*, *z*−1.
